# Draft Genome Sequence of a New Vibrio Strain with the Potential To Produce Bacteriocin-Like Inhibitory Substances, Isolated from the Gut Microflora of Scallop (Argopecten purpuratus)

**DOI:** 10.1128/genomeA.00419-18

**Published:** 2018-05-17

**Authors:** Wilbert Serrano, Ulrike I. Tarazona, Raul M. Olaechea, Michael W. Friedrich

**Affiliations:** a Carrera de Biología Marina, Facultad de Ciencias Veterinarias y Biológicas, Universidad Científica del Sur, Lima, Peru; b Microbial Ecophysiology Group, Faculty of Biology/Chemistry and MARUM, Center for Marine Environmental Sciences, University of Bremen, Bremen, Germany

## Abstract

A new Vibrio strain, V7A, was isolated from the intestinal tract of the Peruvian scallop (Argopecten purpuratus). Strain V7A clusters within the Mediterranei clade of the genus Vibrio and has the potential to produce bacteriocin-like inhibitory substances (BLIS). Here, we report the draft genome sequence of Vibrio mediterranei strain V7A.

## GENOME ANNOUNCEMENT

Vibrios constitute a very successful and versatile group of heterotrophic rod-shaped Gram-negative bacteria known to inhabit a diversity of environments (1, 2). Although some Vibrio species have been collected from pristine waters as free-living organisms (3), they are ubiquitous in marine ecosystems living attached to plankton particles, as symbionts of marine organisms, or associated with marine bivalves (4–6). However, the major concern in the aquaculture industry is pathogenic vibrios, which are deadly for scallop larvae (7). Some species are able to produce bacteriocins, a proteinaceous compound lethal to bacterial species that inhabit the same ecological niche (8). Here, we report the draft genome sequence of Vibrio mediterranei strain V7A isolated from the gut microflora of the Peruvian scallop (Argopecten purpuratus).

As part of the study of the microbiome of A. purpuratus, strain V7A was isolated from fecal pellets extracted from a host organism. Sample collection, enrichment culture, single-colony isolation, genomic DNA extraction, and library preparation for next-generation sequencing (NGS) using the MiSeq sequencer (Illumina) were carried out as described by Serrano et al. (9). The read library contained 789,308 trimmed paired-end reads with an average coverage of 100×. The quality of the reads was determined using the FastQC tool (10), and sequence trimming was performed using Trimmomatic version 0.32 (11). *De novo* assembly was performed using the SPAdes genomic assembler version 3.10.1 (12) and CLC Genomics Workbench 7.0.4 (CLC bio/Qiagen). The draft genome consists of 89 scaffolds with an average length of 63,563 bp. The *N*_50_ value of the assembly was 167,754 bp, with a G+C mol% composition of the DNA of 44.1% and genome size of 5,657,141 bp. Gene prediction and annotation were performed using the online service Rapid Annotations using Subsystems Technology (RAST; http://rast.nmpdr.org) server (13). RAST identified 5,178 coding sequences, of which 1,327 were predicted to encode hypothetical proteins, and 83 predicted noncoding RNAs.

In this report, genomic species circumscription of the new isolate was achieved using the online server JSpeciesWS (14). On the basis of pairwise genome comparison, strain V7A is affiliated with the Vibrio mediterranei clade of the genus Vibrio, with Vibrio mediterranei strain NBRC 15635 as the closest relative. V. mediterranei was isolated from diverse marine environments in the Mediterranean Sea in Spain (15). Some species of V. mediterranei have been reported to produce bacteriocin-like inhibitory substances (BLIS) against the human pathogen Vibrio parahaemolyticus (8), whereas other pathogenic strains are the causative agent of bleaching in the coral Oculina patagonica (16). An average nucleotide identity (ANI) calculation between the genome of strain V7A and other available V. mediterranei genomes from GenBank showed ANI values of 96.74 to 98.99%. These values are clearly above the accepted cutoff value for species delimitation (14). However, strain V7A was the only strain among the examined related Vibrio mediterranei strains showing 24 phage-related gene clusters, among them two types of phage tail proteins, which might be involved in the production of bacteriocins (17). Besides, strain V7A lacks gene clusters involved in the biosynthesis of secondary metabolites, such as polyketide synthase (PKS) or nonribosomal peptide synthetase (NPRS).

### Accession number(s).

This whole-genome shotgun project has been deposited at DDBJ/ENA/GenBank under the accession number PYVE00000000, with the current version PYVE01000000.
